# Immunohistochemical BRAF V600E Expression and Intratumor BRAF V600E Heterogeneity in Acral Melanoma: Implication in Melanoma-Specific Survival

**DOI:** 10.3390/jcm9030690

**Published:** 2020-03-04

**Authors:** Takamichi Ito, Yumiko Kaku-Ito, Maho Murata, Kazuhisa Furue, Che-Hung Shen, Yoshinao Oda, Masutaka Furue

**Affiliations:** 1 Department of Dermatology, Graduate School of Medical Sciences, Kyushu University, Fukuoka 812-8582, Japan; kyumiko@dermatol.med.kyushu-u.ac.jp (Y.K.-I.); muratama@dermatol.med.kyushu-u.ac.jp (M.M.); ffff5113@gmail.com (K.F.); furue@dermatol.med.kyushu-u.ac.jp (M.F.); 2 National Institute of Cancer Research, National Health Research Institutes, Tainan 70456, Taiwan; chshen@nhri.org.tw; 3 Department of Anatomic Pathology, Graduate School of Medical Sciences, Kyushu University, Fukuoka 812-8582, Japan; oda@surgpath.med.kyushu-u.ac.jp

**Keywords:** acral melanoma, BRAF, heterogeneity, immunohistochemistry, mutation, prognosis, outcome, survival, acral lentiginous melanoma, VE1

## Abstract

Acral melanoma, a distinct form of cutaneous melanoma originating in the glabrous skin of the palms, soles, and nail beds, has a different genetic background from other subtypes of cutaneous melanoma. The roles of oncogenic *BRAF* mutations of acral melanoma in pathogenesis and patient outcomes have not been fully elucidated. We retrieved a total of 112 patients with primary acral melanoma and checked their BRAF V600E status using immunohistochemical staining of VE1 antibody. Among these cases, 21 acral melanoma samples (18.8%) showed positive BRAF V600E staining, and of those, 11 samples (9.8%) showed a heterogeneous staining pattern, with a mixture of VE1-positive and VE1-negative cells. BRAF V600E positivity was significantly associated with thicker melanoma (*p* = 0.0015). There was no significant difference in clinicopathological factors between homogeneous and heterogeneous VE1-positive acral melanoma. Both patients with BRAF V600E-positive acral melanoma and those with heterogeneous BRAF V600E had significantly shorter melanoma-specific survival than those with BRAF V600E-negative melanoma in Kaplan–Meier analysis (*p* = 0.0283 and *p* = 0.0065, respectively). These findings provide novel insights into the pathobiology of acral melanoma.

## 1. Introduction

Malignant melanoma, an aggressive malignant tumor derived from melanocytes, occurs in various sites of the body, but most frequently in the skin. Cutaneous malignant melanomas are classified into three subtypes based on the anatomical site and degree of ultraviolet light exposure: melanoma arising from chronically sun-damaged skin, melanoma arising from non-chronically sun-damaged skin, and acral melanoma [1,2]. Many driver gene aberrations have been identified in melanoma, including in *BRAF*, *NRAS*, *NF1*, and *KIT*. These aberrations all activate the mitogen-activated protein kinase (MAPK) pathway, leading to uncontrolled tumor proliferation [2]. Among these driver genes, *BRAF* is the most important in a clinical setting because BRAF/MEK inhibitors can be used for the treatment of *BRAF*-mutated melanoma [3,4]. The latest version of the National Comprehensive Cancer Network (NCCN) guidelines recommends BRAF inhibitors in combination with MEK inhibitors as a first-line treatment for unresectable melanoma with *BRAF* mutations [5].

Acral melanoma is a distinct form of cutaneous melanoma that originates in the glabrous skin of the palms, soles, and nail beds [6,7,8]. It is characterized by a different genetic background from other subtypes and its pathogenesis is less associated with ultraviolet radiation [9,10]. Acral melanoma is rare in Caucasian populations, while it is the most common subtype of melanoma in Asian, African, and Latin American populations [2]. Although several genomic profiling studies of melanoma have been conducted, the numbers of acral melanoma cases in these were limited [11,12,13,14,15]. Furthermore, the genetic heterogeneity of acral melanoma has not been fully investigated. 

We previously investigated our acral melanoma patient cohort and found that the results of immunohistochemistry (IHC) using VE1 antibody, which detects mutated BRAF V600E protein, were highly consistent with the results of an approved commercially available test, the Cobas BRAF V600E Mutation Test (Roche Diagnostics, Mannheim, Germany), and that there was intertumor and intratumor BRAF V600E heterogeneity [16]. In this study, we aimed to further elucidate the roles of BRAF V600E status and BRAF V600E heterogeneity in a larger number of patients with acral melanoma (*n* = 112) and to look at possible associations between these statuses and patient survival.

## 2. Materials and Methods

### 2.1. Ethics Statement

We conducted this investigation in accordance with the concepts enshrined in the Declaration of Helsinki. This study was approved by the Institutional Ethics Committee of Kyushu University (30-363; 27 November 2018).

### 2.2. Patients

We retrieved a total of 112 patients with primary cutaneous acral melanoma who were treated at the Department of Dermatology, Kyushu University, Fukuoka, Japan, between July 2001 and August 2018. For all of these patients, at least three experienced dermatopathologists had confirmed the diagnosis. Clinical and demographic data were retrieved from the patients’ files. Patients were treated in accordance with NCCN melanoma guidelines [5] and only one patient received BRAF/MEK inhibitor treatment during the follow-up period of this study.

Melanoma-specific survival (MSS) and disease-free survival (DFS) were calculated from the date of the first histopathological examination to the date of death as a result of melanoma or the date of recurrence. Data on patients without death or recurrence were censored on the date of the last follow-up, and data on patients who died of other causes were censored at the time of death.

### 2.3. Immunohistochemistry

All formalin-fixed (24 h in 10% buffered formalin) and paraffin-embedded (i.e., formalin-fixed paraffin-embedded (FFPE)) tissues were obtained from our hospital’s archives. IHC staining was performed as reported previously [16,17,18,19,20]. Briefly, the archival FFPE tissue blocks were cut into 4-μm-thick tissue sections and then deparaffinized and rehydrated. Antigen retrieval was performed using Heat Processor Solution pH 9 (Nichirei Biosciences, Tokyo, Japan) at 100 °C for 45 min. The sections were then incubated with mouse monoclonal antibody against human mutated BRAF V600E protein (VE1, ab228461, 1:100; Abcam, Cambridge, UK) or a mouse monoclonal antibody against human Melan A (NCL-L-Melan A, 1:25; Leica Biosystems, Newcastle, UK) at room temperature for 90 min, followed by incubation with an antibody, N-Histofine Simple Stain AP MULTI (Nichirei Biosciences, Tokyo, Japan), for 30 min. Immunoreactions were detected using FastRed II (Nichirei Biosciences, Tokyo, Japan) as a chromogen and were counterstained with hematoxylin. In this method, positive signals of VE1 and Melan A are expressed in red.

For CD68 staining, after retrieving antigen and incubating sections with 3% H_2_O_2_ to block endogenous peroxidase, we used a mouse monoclonal antibody against human CD68 (413791, ready to use; Nichirei Biosciences, Tokyo, Japan) as a primary antibody, N-Histofine Simple Stain MAX-PO MULTI (Nichirei Biosciences, Tokyo, Japan) as a secondary antibody, and 3,3-diaminobenzidine as a chromogen in the same conditions as above. In this method, positive signals of CD68 are expressed in brown. Sections stained without primary antibody served as negative controls.

### 2.4. Evaluation of VE1 IHC Staining

The staining of VE1 in acral melanoma varied in both intensity and proportion of staining-positive cells. We defined tissues as mutation-positive when at least 5% of melanoma cells were positively stained, in accordance with our previous research [16]. The intensity of staining was judged on a semiquantitative scale of 0 to 3+: no staining (0), weakly positive staining (1+), moderately positive staining (2+), and strongly positive staining (3+). Each slide was independently assessed by two dermatopathologists (T.I. and Y.K-I.), who were blinded to the patients’ clinicopathological and mutation data. Slides that received different evaluations by the two observers were again viewed by both observers together, and any discrepancies were resolved by consensus. Heterogeneous staining was defined as the presence in the sample of distinct subpopulations of melanoma cells with both positive and negative immunoreactivities.

As some tumor-infiltrating macrophages may express non-specific positive signals for VE1, we further performed staining with CD68 antibody to distinguish the false-positive staining of macrophages from the true-positive staining of melanoma and carefully assessed the tissues when needed.

### 2.5. Statistical Analysis

All statistical analyses were performed using the JMP Pro statistical software package (version 14.0; SAS, Cary, NC, USA) and the GraphPad Prism statistical software package (version 6; GraphPad Software, San Diego, CA, USA). To evaluate the association between two variables, χ^2^ or Fisher’s exact tests were used as appropriate. The Kaplan–Meier method and the log-rank test were used to evaluate MSS and DFS. For multivariate survival analysis, multivariate Cox proportional hazards regression models were used. A *p* value less than 0.05 was considered to indicate statistical significance.

## 3. Results

### 3.1. Patient Data

We retrieved 112 patients with acral melanoma who were treated at the Department of Dermatology, Kyushu University Hospital, Fukuoka, Japan, between July 2001 and August 2018. Clinicopathological data of all the patients are shown in Table 1. All patients were Japanese, 50 patients (44.6%) were male and 62 (55.4%) female, and the mean age was 65.9 years (range: 16–88). Most of the acral melanomas were histopathologically acral lentiginous melanoma (*n* = 110, 98.2%), while the rest were nodular melanoma (*n* = 2, 1.8%). Among our patients, the primary tumor site was predominantly on the sole (*n* = 68, 60.7%), while cases of acral melanoma on the nail bed in the hand or foot numbered 29 (25.9%). The majority of patients had localized acral melanomas (N0 and M0), but 23.2% of patients had at least one lymph node metastasis (N1–3) and 6.3% had distant metastasis (M1). No patient with in-transit/satellite metastasis without regional lymph node disease (N1c) was noted. 

Among all of the 112 acral melanomas, BRAF V600E was positive in 21 melanomas (18.8%) and negative in the remaining 91 (81.3%), as determined using IHC with a mouse monoclonal VE1 antibody (ab228461; Abcam, Cambridge, UK). VE1 antibody is a BRAF V600E-mutated protein-specific antibody; BRAF V600E positivity was defined as tumors with >5% of positively stained tumor cells, in accordance with our previous study [16]. Interestingly, about half (11 out of 21) of BRAF V600E-positive acral melanomas showed an intratumoral heterogeneous staining pattern (a mixture of positively and negatively stained cells or cell nests). No BRAF V600E-negative acral melanomas showed a heterogeneous staining pattern.

### 3.2. IHC of VE1

**Figure 1:**
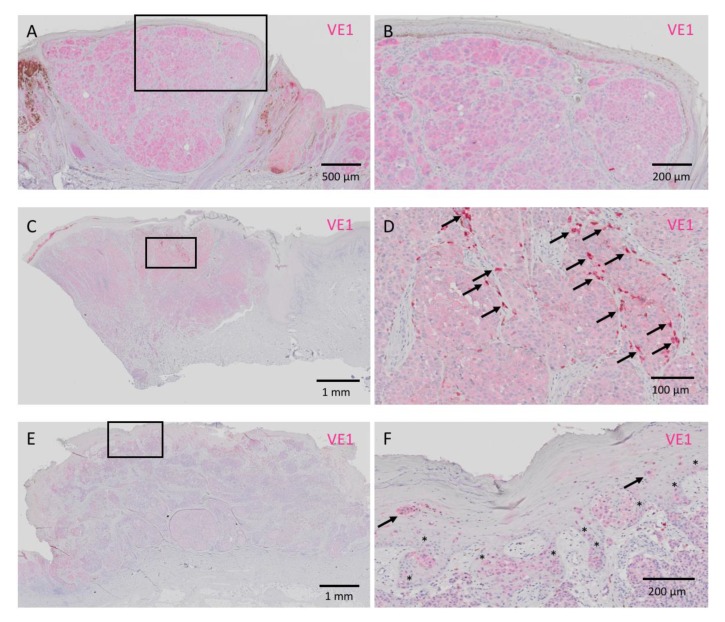
Three cases of acral melanomas with homogeneous VE1-positive staining. (**A**) Strong (3+) VE1 signals are evident. (**B**) High-powered view of the rectangular area in **A**. (**C**) Moderate (2+) VE1 staining of acral melanoma. (**D**) High-powered view of the rectangular area in **C**. Some tumor-infiltrating macrophages showing a strong red color (arrows) are noted. These macrophages should be excluded from the assessment of VE1 staining in melanoma. (**E**) Weak (1+) but definitely positive staining of VE1. (**F**) High-powered view of the rectangular area in **E**. Lentiginous spread of melanoma cells in the basal layer (*) is evident. Ascending melanoma cells (arrows) in the epidermis as well as melanoma cells in the dermis are also VE1 positive. Bars indicate 500 μm in **A**, 200 μm in **B**,**F**, 1 mm in **C**,**E**, and 100 μm in **D**.

**Figure 2:**
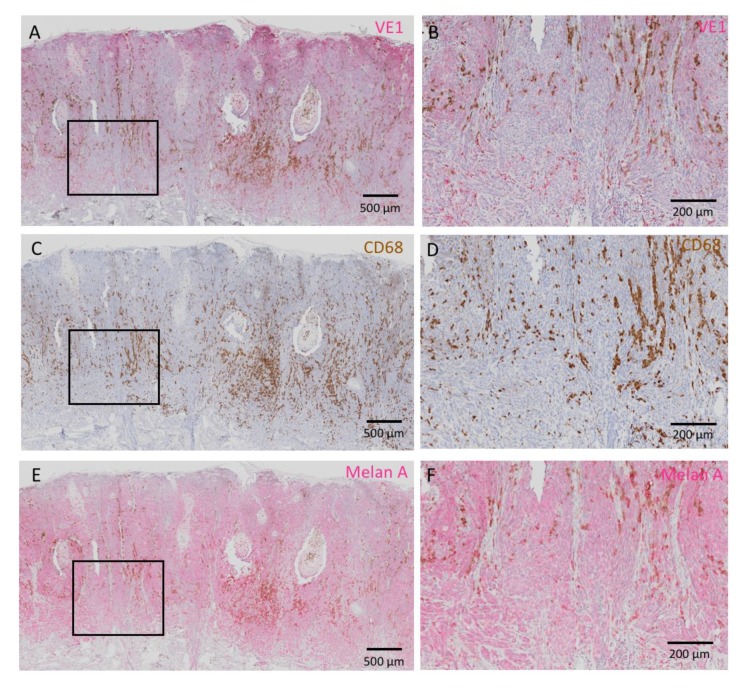
A case of acral melanoma with heterogeneous VE1 staining. VE1-positive signals are shown in red. (**A**) VE1-positive red melanoma cells and VE1-negative melanoma cells are intermingled. (**B**) High-powered view of the rectangular area in **A**. Cells with brown pigments and strongly red cells are scattered. (**C**) CD68 staining of the same area as in **A**. Positive signals of CD68 are shown in brown. (**D**) High-powered view of the rectangular area in **C**. Brown cells in **D** are tumor-infiltrating macrophages. (**E**) Melan A staining of the same area as in **A** and **C**. Positive signals are shown in red. (**F**) High-powered view of the rectangular area in **E**. Melan A staining clearly shows melanoma cells. Bars indicate 500 μm in **A**,**C**,**E**, and 200 μm in **B**,**D**,**F**.

**Figure 3:**
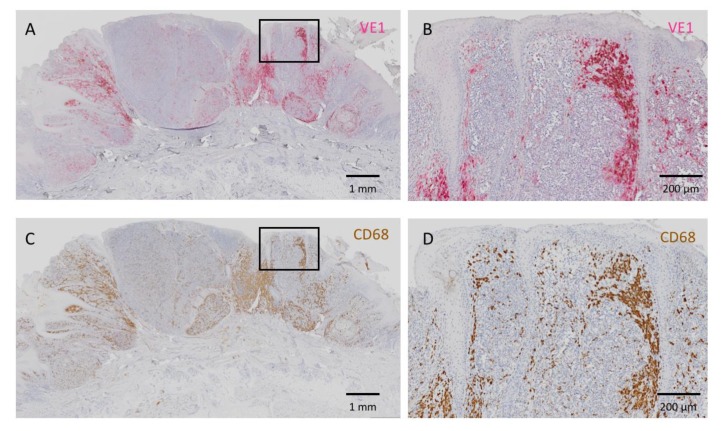
A negative case of VE1 staining. Some cells show strong VE1 expression in red (**A**,**B**), but CD68 staining clearly highlights that these VE1-positive cells are macrophages (**C**,**D**). No melanoma cells stain with VE1 and this case should be regarded as “negative” for VE1. (**B**) High-powered view of the rectangular area in **A**. (**D**) High-powered view of the rectangular area in **C**. Bars indicate 1 mm in **A**,**C** and 200 μm in **B**,**D**.

Representative images of homogeneously positive VE1 staining with different intensities are presented in Figure 1. Positive signals of VE1 were expressed in red. In contrast, an acral melanoma with heterogeneous VE1 expression is shown in Figure 2. In this figure, VE1-positive and -negative cells are intermingled (Figure 2A,B). VE1 antibody sometimes positively reacts with tumor-infiltrating macrophages with a strong staining intensity, and this non-specific staining to macrophages should be carefully differentiated from genuine positive signals of melanoma cells. IHC with CD68, which reacts with macrophages, aids the discrimination of melanoma cells from macrophages (Figure 2C,D). Melan A staining highlights melanoma cells and was relatively homogeneous compared with VE1 staining in this case (Figure 2E,F). Positive signals of VE1 and Melan A are expressed in red and those of CD68 are in brown. Figure 3 shows a VE1-negative acral melanoma. Again, strong red staining of VE1 (Figure 3A,B) was the non-specific type due to reactions with macrophages (Figure 3C,D); such non-specific staining should be regarded as negative as no melanoma cells stain with VE1.

### 3.3. Consistency Between IHC and Real-Time PCR Regarding BRAF V600E Status in Acral Melanoma

Among the 112 acral melanomas, BRAF V600E status was established using Food and Drug Administration (FDA)-approved companion diagnostic kits, the Cobas BRAF V600 Mutation Test (Roche Diagnostics, Mannheim, Germany), or the THxID-BRAF kit (bioMérieux, Lyon, France), in 15 acral melanomas. Real-time PCR was performed in accordance with the manufacturers’ protocols. Three acral melanomas were mutation-positive and 12 were mutation-negative using the kit; these results accorded well with the IHC results, with no discordancy (Supplementary Table S1).

### 3.4. Factors Associated with BRAF V600E Positivity

Table 2 shows the clinicopathological factors associated with BRAF V600E positivity. Among these factors, including the age, sex, primary tumor site, presence of ulceration, T category (thickness of primary tumor), N category (lymph node metastasis), and M category (distant metastasis), only the T category was significantly associated with BRAF status: thick, advanced melanomas (T3 or T4) were more likely to have BRAF V600E-mutated protein (*p* = 0.0015, Fisher’s exact test). No other factors were significantly associated with BRAF V600E mutation.

### 3.5. Comparison with Clinicopathological Factors between Heterogeneous and Homogeneous Acral Melanoma

As about half of BRAF V600E-positive acral melanomas exhibited intratumorally heterogeneous VE1 expression, we next examined which clinicopathological factors were associated with BRAF V600E heterogeneity. Notably, when compared with homogeneously positive BRAF V600E melanoma, heterogeneous BRAF V600 melanoma did not have significant correlation with the following clinicopathological factors: age, sex, primary tumor site, ulceration, T category, N category, and M category, although the number of samples are small (Table 3).

### 3.6. BRAF V600E Positivity is Linked to Worse Patient Survival in Acral Melanoma

**Figure 4:**
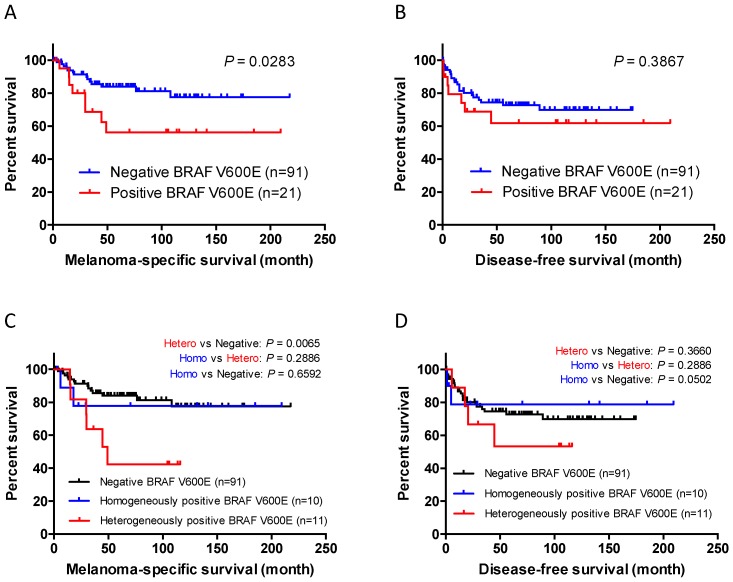
Kaplan–Meier survival curves for melanoma-specific survival (MSS) and disease-free survival (DFS). (**A**) Patients with BRAF V600E-positive acral melanoma had significantly shorter MSS than those with BRAF V600E-negative melanoma (*p* = 0.0283, log-rank test; 5-year MSS, 56.1% and 83.9%, respectively). (**B**) In contrast, BRAF V600E positivity was not a significant prognostic factor for DFS (*p* = 0.3867). (**C**) When BRAF V600E-positive melanoma was divided into heterogeneous and homogeneous subgroups, patients with heterogeneous acral melanomas had worse survival than those with BRAF-negative melanomas (*p* = 0.0065, log-rank test). (**D**) There was no significant difference in DFS among the three subgroups.

As some studies identified *BRAF* mutation as a prognostic factor of melanoma patients, we performed survival analysis using the Kaplan–Meier method. Figure 4 shows the results of the survival curves. Patients with BRAF V600E-positive acral melanoma had significantly shorter melanoma-specific survival (MSS) than those with BRAF V600E-negative melanoma (*p* = 0.0283, log-rank test; 5-year MSS, 56.1% and 83.9%, respectively; Figure 4A). In contrast, BRAF V600E positivity was not a significant prognostic factor for disease-free survival (DFS) in our cohort (*p* = 0.3867, log-rank test; Figure 4B).

We next performed subgroup analyses to further examine the impact of BRAF positivity and BRAF heterogeneity on survival. Interestingly, patients with heterogeneous acral melanomas had worse survival than those with BRAF-negative melanomas (*p* = 0.0065, log-rank test; Figure 4C)**.** However, there was no significant difference in DFS among the three subgroups (Figure 4D).

### 3.7. Cox Multivariate Analysis for Patient Survival

As BRAF V600E positivity was a prognostic factor for MSS in the Kaplan–Meier analysis, we then tested whether it was an independent prognostic factor for MSS and DFS. The results of Cox univariate and multivariate analyses for MSS are shown in Table 4, covering seven variables (age, sex, tumor site, Breslow thickness, ulceration, lymph node metastasis, and BRAF V600E positivity). BRAF V600E positivity and factors with *p* < 0.1 in the univariate analysis were included in the subsequent multivariate analysis. Among these factors, older age, Breslow thickness, ulceration, lymph node metastasis, and BRAF V600E positivity were significant prognostic factors on univariate analysis, and older age and the presence of ulceration retained statistical significance (hazard ratio (HR) 1.06, 95% confidence interval (CI) 1.02–1.18, *p* = 0.0113; and HR 3.82, 95% CI 1.30–11.24, *p* = 0.0148, respectively) on multivariate analysis. Although BRAF V600E positivity had a relatively high HR (1.93), it did not have a statistical significance (*p* = 0.2219).

Regarding DFS, BRAF V600E positivity as well as age, sex, Breslow thickness, ulceration, and lymph node metastasis were included in the multivariate analysis. Male sex (*p* = 0.0154), Breslow thickness (*p* = 0.0043), and lymph node metastasis (*p* = 0.0226) retained statistical significance (Supplementary Table S2). BRAF V600E positivity was not a prognostic factor (*p* = 0.3638).

## 4. Discussion

Similar to many other tumors, malignant melanoma harbors molecular heterogeneity [21,22,23]. Tumor heterogeneity refers to the existence of subclones of tumor cells with distinct molecular variation within individual tumors (intratumor heterogeneity) or among tumors at different sites within a patient (intertumor heterogeneity). In this study, we focused on intratumor BRAF V600E heterogeneity and made several interesting findings. First, we investigated a relatively large number of acral melanoma using immunohistochemical analysis and found that intratumor BRAF V600E heterogeneity was observed in more than half of BRAF V600E-positive melanomas (52.4%, 11/21). Second, both BRAF V600E positivity and BRAF V600E heterogeneity were significantly associated with worse patient survival in the Kaplan–Meier analyses. 

Several DNA-based molecular techniques have been reported to detect *BRAF* gene mutation, including real-time PCR, high-resolution melting, Sanger sequencing, and pyrosequencing [24]. IHC using VE1 antibody is an alternative method for the detection of *BRAF* V600E gene mutation with high concordance with these DNA-based molecular analyses [25,26]. IHC has advantages in that it is less expensive, available in most hospitals as a routine technique, and rapidly yields results. IHC can also be applied to tissues with low tumor content that are not suitable for DNA-based molecular analyses. Another merit of IHC is that it produces clear visualization of the condition of the entire tumor. In this study, we unexpectedly found a high frequency of intratumorally heterogeneous BRAF V600E expression in acral melanoma. In general, *BRAF* gene mutation acts as a driver mutation at the early stage of melanoma development, while subsequent gene aberrations, such as in *TERT, CDKN2A*, and *TP53*, promote the tumor progression at a more advanced stage [2]. Therefore, most cutaneous melanomas have a homogeneous *BRAF* mutation status within a single tumor. Indeed, several studies have found relatively homogeneous BRAF V600E expression in melanoma [27,28,29,30]. However, some studies, including our previous one, have shown definite intratumor heterogeneity of BRAF V600E [16,31,32]. In the current study, we carefully excluded cases of non-specific staining of VE1 due to tumor-infiltrating macrophages and necrosis [33]. CD68 and Melan A were used to highlight the macrophages and melanoma cells, respectively, if necessary (Figure 2 and Figure 3). In suspected cases of heterogeneous VE1 expression, assessment should be performed with caution. The homogeneous and heterogeneous patterns of intratumor BRAF V600E illustrate the complexity and heterogeneity of acral melanoma, which might be due to the mutation of *BRAF* occurring at different stages of acral melanoma progression. 

We also found that patients with acral melanoma exhibiting BRAF V600E staining had significantly poor survival. Oncogenic *BRAF* mutation has been shown to be a factor associated with worse prognosis in cutaneous melanoma. A meta-analysis including 674 melanoma patients showed an increased risk of mortality in patients with *BRAF*-mutated melanoma (HR, 2.25; 95% CI, 1.37–2.12) compared with patients with wild-type *BRAF* melanoma [34]. Another study reported the association of *BRAF* mutation with poorer MSS in melanoma patients [35]. However, the prognostic role of *BRAF* mutation in acral melanoma has not been fully elucidated. The data from this study may reflect the natural history of acral melanoma because only 1 out of 112 patients had received BRAF/MEK inhibitor treatment and the majority of our cohort, thus, had not undergone BRAF-targeted therapy. Intriguingly, when compared to BRAF V600E-negative patients, heterogeneous BRAF V600E acral melanoma patients had worse MSS (Figure 4C) with a significant difference and a small *p* value (*p* = 0.0065), suggesting that heterogeneous acral melanomas, even though they contain BRAF-negative cells in various proportions, are different from BRAF-negative acral melanomas in the tumor biology and are able to act in an aggressive manner. 

Only a few studies have been conducted on the impact of heterogeneous BRAF V600E expression on the clinical response to BRAF/MEK inhibitors so far [31,36]. Wilmott et al. found that neither the staining intensity of VE1 nor heterogeneous staining was associated with outcomes [36]. Another study by Manfredi et al. reported higher progression-free survival in patients with melanoma displaying heterogeneous VE1 staining [31]. Our study differs from these studies in that ours did not investigate the response to BRAF/MEK inhibitors. Investigation on the efficacy of BRAF/MEK inhibitors to heterogeneous acral melanoma will be an interesting future work. The mechanisms by which heterogeneous BRAF V600E expression is associated with worse patient survival are under investigation, and we speculate that acral melanomas with heterogeneous BRAF V600E have similar (or more aggressive) biological behavior to those with homogeneous BRAF V600E.

In conclusion, about half of BRAF V600E-positive acral melanomas had intratumor BRAF V600E heterogeneity. We also found, for the first time, that BRAF V600E positivity and BRAF V600E heterogeneity were significantly associated with worse patient survival in acral melanoma. Further investigation is warranted to examine the possible association between BRAF heterogeneity and the response to BRAF inhibitors in acral melanoma.

## Figures and Tables

**Table 1:** Clinicopathological data of all acral melanoma patients.

Parameters	Number (%)	Parameters	Number (%)
Age in years		N category	
Range (mean ± SD)	16–88 (65.9 ± 15.3)	N0	84 (75.0)
Sex		N1	10 (8.9)
Male	50 (44.6)	N2	7 (6.3)
Female	62 (55.4)	N3	9 (8.0)
Ethnicity		Unknown	2 (1.8)
Japanese	112 (100.0)	M category	
Histopathological subtype		M0	105 (93.8)
Acral lentiginous	110 (98.2)	M1	7 (6.3)
Nodular	2 (1.8)	VE1 staining pattern	
Primary tumor site		Positive	21 (18.8)
Palm	15 (13.4)	Homogeneous	10 (8.9)
Nail bed on hand	17 (15.2)	Heterogeneous	11 (9.8)
Sole	68 (60.7)	Negative	91 (81.3)
Nail bed on foot	12 (10.7)	Detection of BRAF V600E	
Ulceration		IHC	97 (86.6)
Present	43 (38.4)	IHC + real-time PCR	15 (13.4)
Absent	69 (61.6)	MSS in month	
T category		Range (mean ± SD)	1–218 (65.9 ± 51.5)
Tis	29 (25.9)	DFS in month	
T1	23 (20.5)	Range (mean ± SD)	0–209 (59.6 ± 52.8)
T2	10 (8.9)		
T3	17 (15.2)		
T4	33 (29.5)		
Total	112 (100)		

IHC, immunohistochemistry; SD, standard deviation; MSS, melanoma-specific survival; DFS, disease-free survival.

**Table 2:** Factors associated with BRAF V600E positivity.

Parameters	BRAF V600E		*p* Value
	Negative	Positive	
Age in years			
<70	48	13	0.4775
≥70	43	8	
Sex			
Male	38	12	0.2299
Female	53	9	
Primary tumor site			
Palm	11	4	0.1772
Sole	59	9	
Nail bed	21	8	
Ulceration			
Present	35	8	1.0000
Absent	56	13	
T category			
Tis, T1, T2	57	5	0.0015 *
T3, T4	34	16	
N category			
N0	71	13	0.0938
N1–3	18	8	
(Unknown)	(2)		
M category			
M0	85	20	1.0000
M1	6	1	
Total	91	21	

* Significant value. χ^2^ test for primary tumor site and Fisher’s exact test for other parameters.

**Table 3:** Comparison with clinicopathological factors between heterogeneous and homogeneous acral melanoma.

Parameters	Positive BRAF V600E		*p* Value
	Homogeneous	Heterogeneous	
Age in years			
< 70	6	7	1.0000
≥ 70	4	4	
Sex			
Male	5	7	0.6699
Female	5	4	
Primary tumor site			
Palm	2	2	0.4830
Sole	3	6	
Nail bed	5	3	
Ulceration			
Present	3	5	0.6594
Absent	7	6	
T category			
Tis, T1, T2	3	2	0.6391
T3, T4	7	9	
N category			
N0	8	5	0.1827
N1–3	2	6	
M category			
M0	10	10	1.0000
M1	0	1	
Total	10	11	

χ^2^ test for primary tumor site and Fisher’s exact test for other parameters.

**Table 4:** Cox multivariate analysis for melanoma-specific survival.

Variable	Univariate			Multivariate		
	HR	95% CI	*p* Value	HR	95% CI	*p* Value
Age †	1.05	1.02–1.10	0.0040 *	1.06	1.02–1.18	0.0113 *
Male sex	2.24	0.93–5.42	0.0723	2.06	0.78–5.46	0.1449
Tumor site, non-nail bed	2.77	0.81–9.46	0.1031			
Breslow thickness †	1.05	1.02–1.10	0.0053 *	1.13	0.96–1.33	0.1275
Ulceration	6.48	2.49–16.84	0.0001 *	3.82	1.30–11.24	0.0148 *
LN metastasis	4.14	1.69–10.18	0.0019 *	1.06	0.37–3.01	0.9139
BRAF V600E positivity	2.54	1.06–6.05	0.0359 *	1.93	0.67–5.51	0.2219

CI: Confidence interval; HR: hazard ratio; LN: lymph node. † Continuous variables. * Significant values.

## Supplementary Materials

The following is available online at https://www.mdpi.com/2077-0383/9/3/690/s1: Table S1: Cox multivariate analysis for disease-free survival.
