# Supplementary material for: Immunohistochemical BRAF V600E Expression and Intratumor BRAF V600E Heterogeneity in Acral Melanoma: Implication in Melanoma-Specific Survival
Source: J Clin Med. 2020 Mar 4;9(3):690. doi: 10.3390/jcm9030690 (PMC7141227; doi:10.3390/jcm9030690)
Supplement: Supplementary file 1 [file jcm-09-00690-s001.pdf]

Supplementary Table S1: Comparison between PCR and immunohistochemistry in BRAF status

Patients	PCR status	VE1 positive cells (%)	VE1 intensity
1	V600E	100	3+
2	V600E	85	2+
3	V600E	63	2+
4	WT	3	3+
5	WT	3	1+
6	WT	2	2+
7	WT	0	0
8	WT	0	0
9	WT	0	0
10	WT	0	0
11	WT	0	0
12	WT	0	0
13	WT	0	0
14	WT	0	0
15	WT	0	0

Supplementary Table S2: Cox multivariate analysis for disease-free survival

	Univariate			Multivariate		
Variable	HR	95% CI	*P* value	HR	95% CI	*P* value
Age, y†	1.04	1.01–1.07	0.0052*	1.02	0.99–1.05	0.1482
Male sex	2.05	1.04–4.07	0.0389*	2.64	1.20–5.77	0.0154*
Tumor site, non-nail bed	3.37	1.18–9.58	0.0230*			
Breslow thickness†	1.32	1.22–1.44	<0.0001*	1.19	1.06–1.34	0.0043*
Ulceration	7.23	3.34–15.64	<0.0001*	2.30	0.86–6.14	0.0972
LN metastasis	7.45	3.67–15.15	<0.0001*	2.60	1.14–5.89	0.0226*
BRAF V600E positivity	1.39	0.63–3.08	0.4118	1.50	0.62–3.57	0.3638

CI, Confidence interval; HR, hazard ratio; LN, lymph node.

†Continuous variables. *Significant values.
